# Naps in school can enhance the duration of declarative memories learned by adolescents

**DOI:** 10.3389/fnsys.2014.00103

**Published:** 2014-06-03

**Authors:** Nathalia Lemos, Janaina Weissheimer, Sidarta Ribeiro

**Affiliations:** ^1^Laboratory of Memory, Sleep and Dreams, Brain Institute, Federal University of Rio Grande do NorteNatal, Brazil; ^2^Department of Physiology, Psychobiology Graduate Program, Federal University of Rio Grande do NorteNatal, Brazil; ^3^Department of Foreign Languages and Literatures, Federal University of Rio Grande do NorteNatal, Brazil; ^4^ACERTA Program, Education Observatory CAPES/INEPNatal, Brazil

**Keywords:** learning and memory, sleep, memory consolidation, middle school

## Abstract

Sleep helps the consolidation of declarative memories in the laboratory, but the pro-mnemonic effect of daytime naps in schools is yet to be fully characterized. While a few studies indicate that sleep can indeed benefit school learning, it remains unclear how best to use it. Here we set out to evaluate the influence of daytime naps on the duration of declarative memories learned in school by students of 10–15 years old. A total of 584 students from 6th grade were investigated. Students within a regular classroom were exposed to a 15-min lecture on new declarative contents, absent from the standard curriculum for this age group. The students were then randomly sorted into nap and non-nap groups. Students in the nap group were conducted to a quiet room with mats, received sleep masks and were invited to sleep. At the same time, students in the non-nap group attended regular school classes given by their usual teacher (Experiment I), or English classes given by another experimenter (Experiment II). These 2 versions of the study differed in a number of ways. In Experiment I (*n* = 371), students were pre-tested on lecture-related contents before the lecture, were invited to nap for up to 2 h, and after 1, 2, or 5 days received surprise tests with similar content but different wording and question order. In Experiment II (*n* = 213), students were invited to nap for up to 50 min (duration of a regular class); surprise tests were applied immediately after the lecture, and repeated after 5, 30, or 110 days. Experiment I showed a significant ~10% gain in test scores for both nap and non-nap groups 1 day after learning, in comparison with pre-test scores. This gain was sustained in the nap group after 2 and 5 days, but in the non-nap group it decayed completely after 5 days. In Experiment II, the nap group showed significantly higher scores than the non-nap group at all times tested, thus precluding specific conclusions. The results suggest that sleep can be used to enhance the duration of memory contents learned in school.

## Introduction

There is an increasing interest in understanding the importance of sleep for academic performance in children and adolescents. The transition to adolescence comprises marked changes in sleep-wake patterns and underlying physiological factors, including typical behaviors such as delayed sleep phase syndrome (Gianotti et al., 1992; Louzada and Menna-Barreto, 2003, 2004; Crowley et al., 2007; Sousa et al., 2007; Beijamini and Louzada, 2012). In particular, eveningness is associated with later bedtime and wake-up time (especially on weekends), shorter time in bed during the week, longer weekend time in bed, irregular sleep-wake schedule, and subjective poor sleep (Crowley et al., 2007). Delayed phase syndrome is associated with extrinsic and intrinsic factors, such as electricity at home, technology and social context, as well as a strong relationship with pubertal maturation during development (Carskadon et al., 1993; Louzada and Menna-Barreto, 2003, 2004; Sousa et al., 2009). Together, delayed sleep phase syndrome, sleep habits and early starting times for school are responsible for promoting sleep deprivation in adolescents; and consequently, increase daytime sleepiness, attention and emotional problems, difficulties in memorization and concentration, and poor school achievement (Gianotti et al., 1992; Sousa et al., 2007; Beijamini and Louzada, 2012). This situation is not very different when it comes to children, whose typical school times and extracurricular activities reduce opportunities for daytime naps that are common among preschoolers. Furthermore, inadequate sleep in children has been related to attention deficits, irritability, emotional fragility and frustration (Dahl, 1996; Belísio et al., 2010). Changes in the sleep-wake cycle are thought to jeopardize school learning in two ways: they reduce the capacity for new learning, and impair the consolidation of what has already been learned (Louzada et al., 2008).

Some studies suggest negative health effects of napping among adults, such as a link to higher risk of diabetes, with different nap durations having different effects on health (Fang et al., 2013; Lucassen, 2013). On the other hand, multiple laboratory studies indicate that sleep enhances neural plasticity and increments learning and memory (Maquet, 2001; Stickgold, 2005; Born et al., 2006; Walker and Stickgold, 2006; Ellenbogen et al., 2009; Diekelmann and Born, 2010). Declarative memories are specifically enhanced by slow wave sleep in both adults (Yaroush et al., 1971; Fowler et al., 1973; Plihal and Born, 1997) and children (Wilhelm et al., 2008; Prehn-Kristensen et al., 2009). Since slow wave sleep comprises most part of daytime naps (Bes et al., 1996), and declarative memories constitute the bulk of what is learned in school, naps have great potential to benefit school learning. In adults, daytime naps of 60–90 min enhance perceptual learning nearly to the same extent as an 8 h period of night time sleep (Mednick et al., 2003), and naps as short as 6 min have been reported to benefit declarative memory retention (Lahl et al., 2008). In infants, naps have been shown to enhance language learning (Gomez et al., 2006; Hupbach et al., 2009).

Despite the heightened interest on the cognitive role of naps, the actual effectiveness of naps in school learning remains largely unexplored. A recent study showed that school naps enhance learning in preschoolers (Kurdziel et al., 2013), but no information about adolescents is available. In the present study we investigated whether post-learning daytime naps can benefit the consolidation of declarative memories learned in school by adolescents, with a focus on a potential effect on memory duration.

## Methods

### Subjects

A total of 584 volunteer students (10–15 years old, mean age 11.3) from the 6th grade participated in the study, which involved 7 schools in the Brazilian city of Natal. The volunteers were healthy, did not present sleep disturbances, and did not use medication that affects the sleep/wake cycle. Students that failed any of those requirements were excluded from the analysis. The parents or legal guardians of the students signed consent forms with a description of the study procedures. The study was approved by the Research and Ethics Committee of the Onofre Lopes Hospital at the Federal University of Rio Grande do Norte (permit 336/09).

### General experimental design

**Figure 1:**
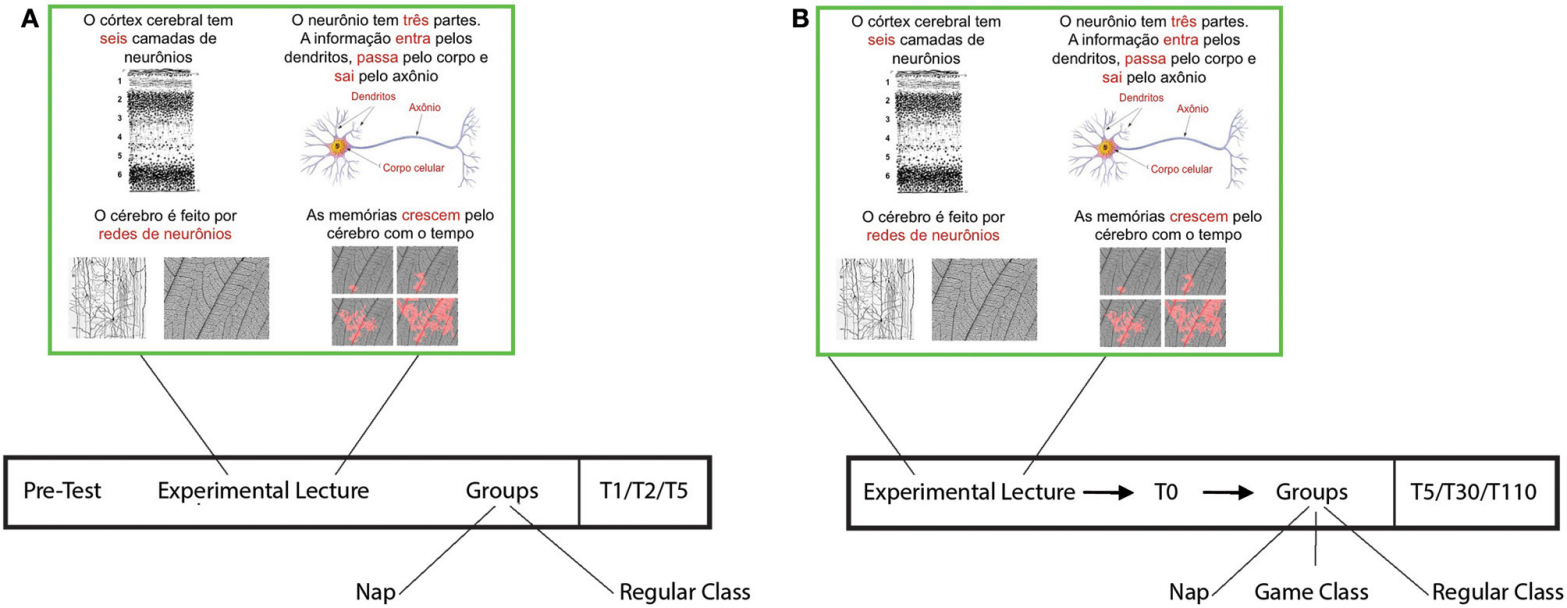
**Experimental design**. Students were exposed to a lecture on novel declarative contents, sorted to nap or not to nap, and subjected to surprise tests after 1, 2, and 5 days. **(A)** Experiment I. **(B)** Experiment II. ^*^Indicates significant differences with respect to the pre-test.

Data collection took place entirely at school, without adjustment for the amount of sleep obtained by each student at home. In the morning (between 08:00 and 09:00) or in the afternoon (between 14:00 and 15:00) the students were exposed to a 15-min lecture on “vision and memory,” a content that is typically unknown by the age group studied, and is absent from the standard curriculum of the 6th grade. The contents of the lecture were intentionally designed not to be relevant to the curriculum, thus avoiding interference with memories acquired before or during the experiment. The lecture was always presented by the same experimenter (NL) as a fixed sequence of images plus text projected on a screen. From a pedagogical point of view, the experimental lectures were designed to be highly engaging to the students in two ways: (i) the researcher (NL) always activated the students' schemata and elicited their prior knowledge, so that they could link it to the new input in an active fashion; (ii) the lecture was technology-mediated (a video projector was used) and attractive (high-resolution images of high perceptual salience), a rare practice in Brazilian public schools which may explain why the students typically reacted with enthusiasm and sometimes euphoria to the presence of the experimenter (NL). Immediately after the lecture, the students were randomly sorted into nap and non-nap groups. Students in the nap group were conducted to a quiet room with mats, received sleep masks and were invited to sleep. Students in the non-nap group proceeded to different interfering activities (see below). Finally, all the students were allowed to return to their regular classroom activities (Figure 1). The experimenter (NL) questioned subjects after their nap interval on whether they had slept, and/or dreamed. However, due to the degree of subjectivity and lack of reliability of this measure, these data were not included in the analyses and we decided to place all participants with access to sleep in the nap condition. Tests were administered without prior notice to the students at the same time of the lecture. Each student took two tests applied on different days. We did not seek to determine whether students studied the material at home, for we considered self-reporting too subjective. However, we took three measures to discourage the students to look further into the topics: students were not allowed to take notes during the lecture, the topics were off the curriculum, and the students were not aware that the researchers would come back days later to apply a test.

### Experiment I

Students in the nap group were invited to nap for up to 2 h, and students in the non-nap group received a regular school class given by their regular teacher. A multiple-choice pre-test (PT; Supplementary Material 1) for lecture-related knowledge was applied immediately before the lecture, and a test comprising similar content but different in wording and question order was applied after 1, 2, or 5 days (T1, T2 and T5, respectively; Supplementary Material 2; Figure 1A). Therefore, each student was tested twice.

### Experiment II

Students in the nap group were invited to nap for up to 50 min, which is the duration of a regular class period. The non-nap condition consisted of two sub-groups of equal size: one attended a regular English class given by their usual teacher; and another participated in an English class with games given by another experimenter (JW). A multiple-choice test was applied immediately after the lecture (T0), and identical follow-up tests were applied after 5, 30 or 110 days (T5, T30, or T110, respectively; Figure 1B; Supplementary Material 2). Therefore, each student was tested twice. Participants whose T0 scores were lower than 50% were discarded from the analysis.

### Statistical analyses

Data from morning and afternoon tests were pooled. Significant differences were assessed using ANOVA followed by two-tailed Student's *t*-tests. One-way ANOVAs were used to test, within each contrast assessed (e.g., Experiment 2, T0 vs. T5) whether scores were higher when lectures were followed by naps, in comparison with waking activities. Two-Way ANOVAs were used to search for interactions between sleep (nap vs. no-nap) and time of the day (morning vs. afternoon) with regard to test scores, as well as interactions between sleep (nap vs. no-nap) and time of testing (T0, T5, and T30) with regard to memory deterioration.

## Results

**Figure 2:**
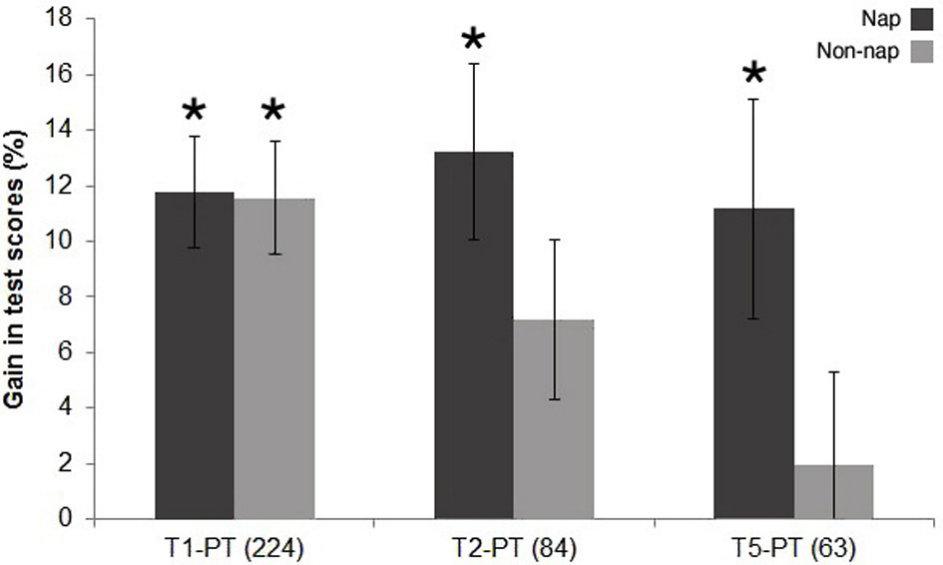
**Experiment I—Gains in tests scores between pre- and post-tests applied at 1, 2, or 5 days after learning**. Nap (*N* = 191, black bars) and non-nap (*N* = 180, gray bars) groups. Numbers in parenthesis indicate *N* assessed at each post-test day. Asterisk for *P* < 0.05.

A total of 371 students (mean age 11.5 years old) participated in Experiment I. Figure 2 illustrates score gains for both the nap and non-nap groups, calculated as the difference between post-class (T1, T2 ou T5) and pre-class (PT) scores. Both groups showed a significant gain in test scores from pre-test to T1: nap (11.7 ± 2.0, mean % ± s.e.m., *P* < 0.0001) and non-nap (11.5 ± 2.0, mean % ± s.e.m., *P* < 0.0001). This gain was sustained in the nap group for T2 (13.2 ± 3.1, mean % ± s.e.m., *P* < 0.0001) and T5 (11.1 ± 3.9, mean % ± s.e.m., *P* = 0.016), but in the non-nap group it halved at T2 (7.1 ± 2.8, mean % ± s.e.m., *P* = 0.039) and decayed completely at T5 (1.9 ± 3.3, mean % ± s.e.m., *P* = 0.568) in the non-nap group.

**Figure 3:**
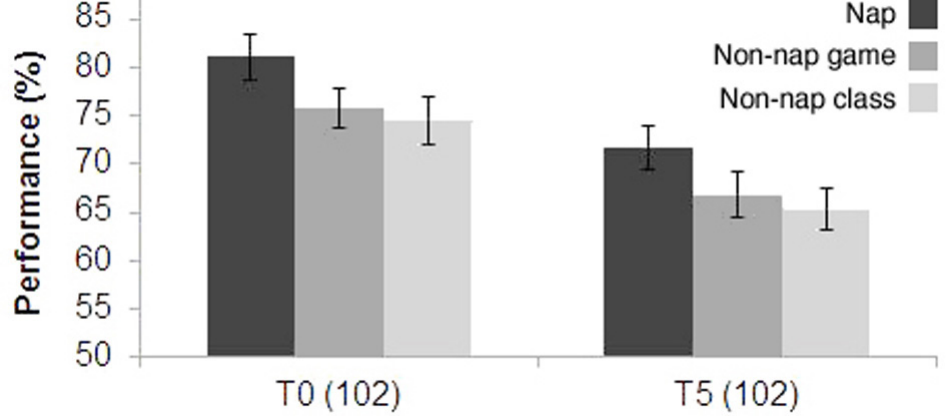
**Experiment II—Test performance immediately after the lecture (T0), and 5 days later (T5)**. Nap (*N* = 32), non-nap groups for game class (*N* = 41), and non-nap groups for regular class (*N* = 29).

A total of 213 students (mean age 11.0 years old) participated in Experiment II. Figure 3 shows performance 5 days after class (T5) in the three conditions: nap, non-nap with regular English class, and non-nap with game class in English. Performance was calculated as the ratio between the number of correct answers and the total number of questions in the test. All the three groups in Experiment II displayed a significant decrease in test scores after 5 days (*P* = 0.006): 71.7 ± 2.2 (mean % ± s.e.m.) for the nap group; 66.8 ± 2.3 (mean % ± s.e.m.) for the game class group; and 65.3 ± 2.0 (mean % ± s.e.m.) for the regular class group. A significant difference was found exclusively among the nap and regular class groups (*P* = 0.044); however, T0 already indicated a trend toward this difference between the two groups (*P* = 0.055).

**Figure 4:**
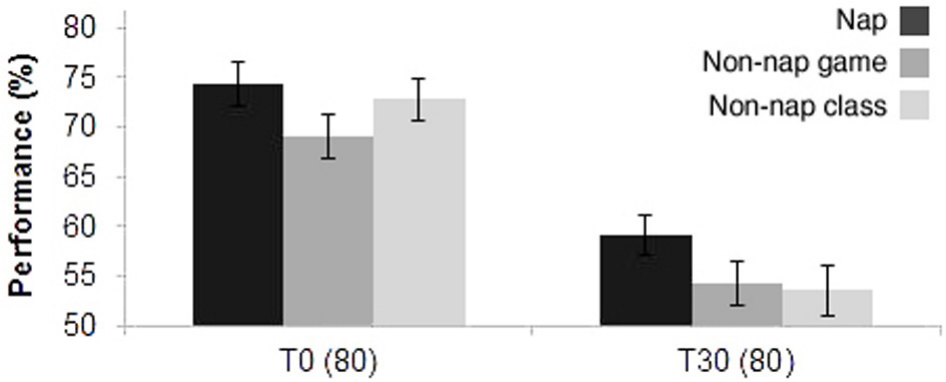
**Experiment II—Test performance immediately after the lecture (T0), and 30 days later (T30)**. Nap (*N* = 23), non-nap groups for game class (*N* = 30), and non-nap groups for regular class (*N* = 27).

Figure 4 displays the performance of the groups assessed at T0 and T30. As expected, the three groups displayed a significant decrease in test performance after 30 days (*P* < 0.0001): 59.1 ± 1.9 (mean % ± s.e.m.) for the nap group; 54.3 ± 2.2 (mean % ± s.e.m.) for the game class group; and 53.5 ± 2.4 (mean % ± s.e.m.) for the regular class group. No significant differences were found between the groups within each test day (T0 and T30). However, T30 scores revealed a statistical trend for higher performance in the nap group, in comparison with the non-nap class group (*P* = 0.090).

**Figure 5:**
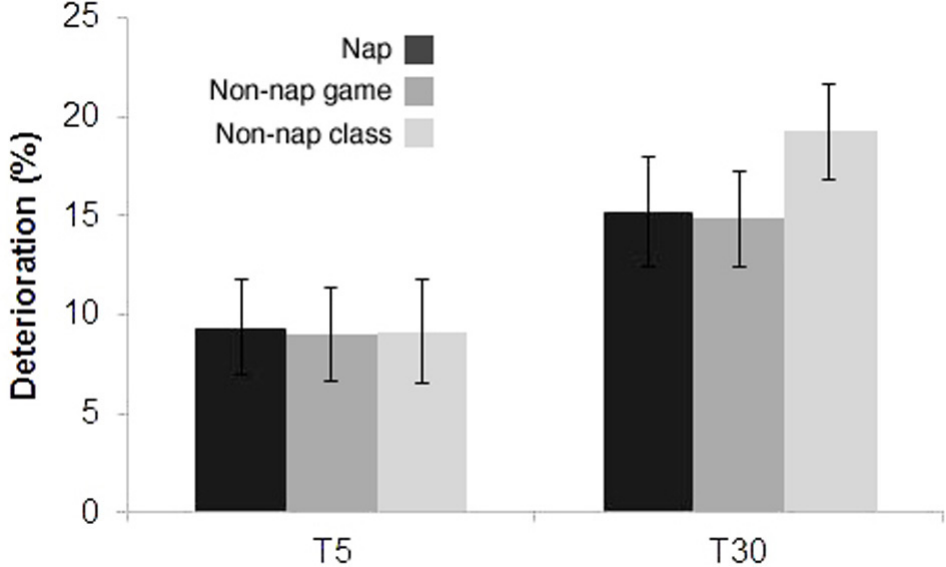
**Experiment II—Learning deterioration over time**. Difference in performance, from T0 to T5 or T30.

Figure 5 shows the deterioration in performance, from T0 to T5 or T30. At T5, we found a decrease of 9.3 ± 2.3 (mean % ± s.e.m.) for the nap group; 9.0 ± 2.3 (mean % ± s.e.m.) for the game class group; and 9.1 ± 2.6 (mean % ± s.e.m.) for the regular class group. At T30, we found a decrease of 15.2 ± 2.7 (mean % ± s.e.m.) for the nap group; 14.8 ± 2.4 (mean % ± s.e.m.) for the game class group; and 19.2 ± 2.4 (mean % ± s.e.m.) for the regular class group. A two-way ANOVA detected a significant effect of time (*P* = 0.0006) but no significant difference across the T0, T5 and T30 groups (*P* = 0.6296), nor a significant interaction between time and groups (*P* = 0.6193). At T110, no group showed significant differences from chance performance (*N* = 31 per group). Across all groups, there were no significant differences in the time that students reported to have slept in the night prior to the tests.

## Discussion

We investigated whether daytime naps can benefit declarative learning by adolescents in the school setting. The results of Experiment I suggest that the benefits of a nap taken immediately after school learning remain stable for at least 5 days after the initial learning. These benefits cannot be attributed to putative attention deficits due to less sleep in the non-nap group, since the surprise tests were always applied after at least one full night of sleep. Therefore, the recently acquired memories must have been positively impacted by the nap itself. The results suggest that the lecture-related memories in the non-nap group were more fragile than in the nap group, and a full night of sleep many hours after learning was not enough to compensate for such fragility. The results in Experiment II were inconclusive, because the differences between the nap and non-nap groups were already present at T0, and did not change in proportion over time. While the results of Experiment I indicate that daytime nap can be used to enhance school learning, the results in Experiment II demand a better controlled replication.

Experiments I and II were not designed to test the effect of a single variable of interest, but rather represented two separate attempts to assess the cognitive potential of naps in the school setting, under quite different constraints. A major difference between the two experiments was the length of nap allowed. While Experiment I targeted a 2 h interval, which provides ample opportunity for napping and even for traversing a complete sleep-wake cycle, Experiment II conformed to the standard 50 min per class that is the norm in Brazilian schools. Therefore, differences in nap length may well account for the differences in the results obtained from the two experiments. In that respect, an important limitation of our study is the fact that the students were not recorded with an actimeter or an electroencephalograph, which would have provided quantitative information about sleep amount and the relative contribution of different sleep stages.

In Experiment I, baseline (pre-lecture) knowledge was measured and then each student was subjected to a surprise post-lecture test with highly overlapping content but shuffled options for multiple responses (Supplementary Materials 1, 2). The rationale for this testing scheme was to avoid habituation to the same stimuli, mimicking a pedagogic procedure often employed by teachers to assess learning over time. The lack of testing immediately after the lecture aimed to avoid a “testing effect” prior to consolidation (Carrier and Pashler, 1992; Roediger and Karpicke, 2006). The design of Experiment II, in contrast with Experiment I, assessed exactly the same information immediately post-lecture and again after 5, 30 or 110 days, allowing for a direct measurement of memory retention. Pre-testing was not performed in this case to avoid a ceiling effect at T0. Neither experiment measured knowledge acquisition specifically across the learning experience, making it impossible to determine whether new learning occurred or not during the lecture. Yet, both designs assessed the relationship between post-lecture sleep and memory retention, with reference to pre- and post-lecture levels, for designs I and II respectively. Experiments I and II differed by two variables with respect to testing, and additional experiments would be necessary to determine the effects of testing immediately before or after the lecture, an aspect beyond the scope of this work. Since 2009 we initiated several experiments related to those described in this study, but most were botched due to the frequent occurrence of teacher strikes in Brazilian schools. In Experiment II, the group assessed after 110 days was originally intended to be tested after 30 days, but a strike intervened. In that sense, the design differences in Experiments I and II, without additional experiments able to separately test all the variables involved, stem from the tentative dynamics of classroom research in Brazil. The difficulties we faced likely apply to classroom research in most of the developing world.

Data were collected both in the morning and in the afternoon for practical reasons: In the Brazilian state of Rio Grande do Norte, where the study was carried out, some schools offer 6th grade classes in the morning but not in the afternoon and vice-versa, because students stay in school only half a day. Although this introduced an additional variable in the study, we found no statistical interaction (*P* = 0.18) between time (morning and afternoon) and condition (nap vs. no nap), in line with previously published data showing equivalent sleepiness at 08:00 and 14:00 among Rio Grande do Norte adolescent students during school days (Sousa et al., 2009). For this reasons, morning and afternoon data were pooled.

It is important to note that sleep is also involved in forgetting (Rolls et al., 2013; Atherton et al., 2014; Oudiette et al., 2014). The present study was specifically aimed at assessing the role of sleep in memory retention, but it was not designed to address forgetting. Studies which set out to investigate the role of sleep in forgetting usually have a distinct paradigm, i.e., they often cue participants in the encoding phase either to remember or forget specific information after sleep. For instance, Saletin et al. (2011) demonstrated that sleep, relative to time spent in waking, can selectively enhance the recall for words previously cued for remembering. In contrast, no such facilitation was observed for items tagged for forgetting. In our study the participants were not cued for remembering nor forgetting, since they were not aware of the post-learning test. Therefore, the issue of forgetting could not be directly addressed by our research design.

Social jetlag plays a key role in the health and functioning of adolescents. Delaying the time of school onset for adolescents and allowing them to nap between classes are relatively easy implementations with potential to reduce classroom sleepiness in a cost-effective manner, thus representing “low-hanging fruits” in the application of neuroscience findings to school education (Ribeiro and Stickgold, 2014; Sigman et al., 2014). School learning is a complex cognitive endeavor, influenced by a myriad of factors beyond physiology, such as the teachers' motivation to teach, the students' intrinsic and extrinsic motivations to learn; the teacher's level of education; the degree of family support etc. Despite all this complexity, the experiments reported here support the use of daytime naps to enhance the duration of declarative contents learned in school. One problem that needs to be addressed is that the use of naps to benefit school learning would likely decrease the time spent in classes undergoing formal exposure to novel and old contents. To prevent a negative impact on school curriculum, the time and amount of post-learning naps must be optimized.

## Author contributions

Sidarta Ribeiro and Nathalia Lemos conceived and designed the experiments; Nathalia Lemos, Janaina Weissheimer and Sidarta Ribeiro performed the experiments; Nathalia Lemos and Sidarta Ribeiro analyzed the data; Sidarta Ribeiro, Nathalia Lemos and Janaina Weissheimer wrote the paper.

### Conflict of interest statement

The authors declare that the research was conducted in the absence of any commercial or financial relationships that could be construed as a potential conflict of interest.
