# Supplementary material for: Naps in school can enhance the duration of declarative memories learned by adolescents
Source: Front Syst Neurosci. 2014 Jun 3;8:103. doi: 10.3389/fnsys.2014.00103 (PMC4042263; doi:10.3389/fnsys.2014.00103)
Supplement: Supplementary file 1 [file DataSheet1.PDF]

NAME:_________________________________ AGE:_____ GENDER:_____
SCHOOL:_______________________________ GRADE:_____

1. What is the colored part of the eye called?
( ) Tiris
( ) Eyebrow
( ) Iris
( ) Fovea

2. What organ is usually referred to as the “WINDOW” of the human body?
( ) Ear
( ) Eye
( ) Skin
( ) Liver

3. How many parts does a neuron have?
( ) 1
( ) 2
( ) 3
( ) 4

4. What is the white part of our eyes called?
( ) Iris
( ) Eyelashes
( ) Retina
( ) Sclera

5. What tool do we use to amplify images?
( ) Drill
( ) Metroscope
( ) Stetoscope
( ) Microscope

6. Which animal has hundreds of mini eyes?
( ) Alligator
( ) Dolphin
( ) Fly
( ) Bat

7.What are these mini eyes called?
( ) Osmatideos
( ) Ommenideos
( ) Ommatidia
( ) Ostramondra
8. Where in the eye does light come in through?
( ) Pupil

( ) Retina
( ) Optic Nerve
( ) Cortex

9. What is the purpose of the retina?
( ) To capture the image in the eye
( ) To color the image in the eye
( ) To enlarge the image in the eye
( ) To shorten the image in the eye

10. What is the “HALL of vision” called?
( ) Optic Nerve
( ) Halo Nerve
( ) Tibial Nerve
( ) Auditory Nerve

11. What part involved in the processing of the visual images we capture is named after “BED”?
( ) Cortex
( ) Optic Nerve
( ) Thalamus
( ) Skull

12. What is the external part of the brain (the CRUST) called?
( ) Cortex
( ) Optic Nerve
( ) Skull
( ) Thalamus

13. Where does information come in through the neuron?
( ) Dendrites
( ) Axon
( ) Galaxy
( ) Cell body

14.Where does information exit the neuron through?
( ) Dendrites
( ) Axon
( ) Nucleus
( ) Planet

15.How many parts is the cortex made of?
( ) 3
( ) 4
( ) 5
( ) 6

16. What are the stripes that can be seen in the monkey’s visual cortex called (when one of its eye is closed and the other one is open) ?
( ) Ocular absence columns
( ) Ocular dominance columns
( ) Ocular miniature columns
( ) Ocular representation columns

17. What grows in the brain with time?
( ) Eyes
( ) Thalamus
( ) Cortex
( ) Memory

18. What are the two routes in the human visual cortex called?
( ) Dorsal and Ventral
( ) Vertical and Horizontal
( ) Medial and Horizontal
( ) Initial and Final

19. What is the route responsible for the localization of the object?
( ) Lateral
( ) Medial
( ) Ventral
( ) Dorsal

20. The brain is made of:
( ) No neurons
( ) A single neuron
( ) Two neurons
( ) Neural networks
