# Supplementary material for: Naps in school can enhance the duration of declarative memories learned by adolescents
Source: Front Syst Neurosci. 2014 Jun 3;8:103. doi: 10.3389/fnsys.2014.00103 (PMC4042263; doi:10.3389/fnsys.2014.00103)
Supplement: Supplementary file 2 [file DataSheet2.PDF]

NAME:_________________________________ AGE:_____ GENDER:_____
SCHOOL:_______________________________ GRADE:_____

1. What part of the human body resembles a “window”?
( ) Ear
( ) Eyebrow
( ) Mouth
( ) Eye

2. A human being has two eyes. How many mini eyes does a fly have?
( ) Hundreds
( ) Three
( ) Thousands
( ) Dozens

3. “Ommatidea” is what we call which animal’s eyes?
( ) Fish
( ) Frog
( ) Alligator
( ) Fly

4. The human eye consists of the following parts:
( ) Iris, sclera and pupil
( ) Iris, retina and coclea
( ) Retina, optic nerve and larynx
( ) Esophagus, cornea and sclera

5. What is the nerve that transports the visual stimuli called?
( ) Halo nerve
( ) Optic nerve
( ) Vague nerve
( ) Trigeminal nerve

6. What is the iris responsible for?
( ) It controls the light entrance
( ) It is the colored part of the eye
( ) It grows the hair in the eyelids
( ) It is the white part of the eye

7. What structure is responsible for capturing the image in the eye?
( ) Pupil
( ) Crystalline lens
( ) Retina
( ) Sclera
8.The thalamus can be compared to a:
( ) window

( ) table
( ) bed
( ) trunk

9. Which structure can we compare to a “crust”?
( ) Auditory cortex
( ) Pupil
( ) Visual cortex
( ) Fovea

10. A neuron consists of the following parts:
( ) Axon, dendrites and optic nerve
( ) Sclera, dendrites and pupil
( ) Iris, axon and fovea
( ) Dendrites, cell body and axon

11. The cortex has six layers of:
( ) Glias
( ) Neurons
( ) Cornea
( ) Dendrites

12. Through a microscope we can see structures which are:
( ) Transparent
( ) Larger
( ) Non-existent
( ) Smaller

13.There is a greater number of neurons in the following layers:
( ) 2 and 5
( ) 3 and 4
( ) 2 and 4
( ) 4 and 5

14. What are the columns in the visual cortex called?
( ) Slim
( ) Small
( ) Thick
( ) Dominance

15. When we close one eye and leave the other one open, what image can we see in the visual cortex?
( ) Triangles

( ) Dots
( ) Squares
( ) Stripes

16.What happens to our memories in the brain through time?
( ) They diminish
( ) They remain the same
( ) They vanish
( ) They grow

17. How many routes are there in the human visual cortex?
( ) 1
( ) 2
( ) 3
( ) 4

18. An object’s identity is determined by which route?
( ) Lateral
( ) Dorsal
( ) Final
( ) Ventral

19. The brain is made of:
( ) no neurons
( ) a single neuron
( ) two neurons
( ) neural networks

20. What do the fly, the alligator and the human being have in common?
( ) Teeth
( ) Wings
( ) Memories
( ) Nails
